# The course of acute low back pain: a community-based inception cohort study

**DOI:** 10.1097/PR9.0000000000001152

**Published:** 2024-04-10

**Authors:** Fabian Pfeiffer, Hannu Luomajoki, André Meichtry, Sabina Hotz Boendermaker

**Affiliations:** a Zurich University of Applied Sciences, School of Health Sciences, Institute of Physiotherapy, Winterthur, Switzerland; b School of Health Professions, Bern University of Applied Sciences, Bern, Switzerland; c Pain in Motion Research Group, Department of Physiotherapy, Faculty of Physical Education and Physiotherapy, Vrije Universiteit Brussel, Belgium

**Keywords:** Low back pain, Natural course, Trajectory, Latent class linear mixed model

## Abstract

**Introduction:**

Acute low back pain (LBP) is increasingly recognized for its potential recurrent nature and long-term implications.

**Objectives:**

This community-based inception cohort study aimed to delineate trajectories of acute LBP over one year and investigate associated biopsychosocial variables.

**Methods:**

One hundred seventy-six participants with acute LBP were monitored at 5 follow-up time points over 52 weeks. Pain trajectories were identified using a latent class linear mixed model, and their associations with baseline biopsychosocial factors were evaluated through multinomial logistic regression.

**Results:**

Four distinct LBP trajectories were discerned: “mild/moderate fluctuating pain” (54.0%), “delayed recovery by week 52” (6.2%), “persistent moderate pain” (33.0%), and “moderate/severe fluctuating pain” (6.8%). Increased baseline pain intensity and history of LBP episodes were significantly linked with less favorable trajectories. Contrary to expectations, psychological variables like stress, anxiety, and depression did not significantly associate with unfavorable trajectories.

**Discussion:**

This study underscores the heterogeneity of acute LBP's course over a year, challenging the conventionally benign perception of the condition. Recognizing these distinct trajectories might enable more tailored, effective clinical interventions for LBP patients. The small sample size of certain trajectories may influence the generalizability of the results.

**Conclusion:**

Acute LBP can manifest in different trajectories, with nearly half of the participants experiencing less favorable trajectories. Baseline pain intensity and previous episodes of LBP emerged as key factors, whereas psychological variables had no discernible influence. Recognition of these trajectories may be necessary for improved patient management and targeted interventions.

## Abstract 2

Supplemental Digital Content is Available in the Text.

One-year acute low back pain trajectories are determined by initial pain intensity and past episodes, not by psychological factors.

## 1. Introduction

Low back pain (LBP) is an increasingly prevalent musculoskeletal disorder, with a lifetime prevalence of 84%.^38^ Low back pain was responsible for 63.7 million years of living with disability worldwide in 2019.^3^ Serious pathologies or specific causes contribute only 10% of LBP presentations.^18^ Most LBP presentations are nonspecific, a reflection of the intricate interplay of biopsychosocial factors and comorbidities.^11^

The management of LBP is a major challenge in clinical practice due to its recurrent nature, varying pain intensities, and different biopsychosocial factors. Historically, the approach to LBP management has relied on a temporal classification system that categorizes pain into acute (less than 6 weeks), subacute (6–12 weeks), and chronic (more than 12 weeks) phases.^36^ Initially, this classification system was based on the assumption that acute, nonspecific LBP leads to significant recovery shortly after onset.^4^ Understanding the heterogeneity of LBP presentations necessitates a shift away from traditional classification systems.^19^ It calls for more individualized approaches that can accurately reflect the nonlinear and variable trajectories acute LBP can undertake over time,^9,19^ with many individuals experiencing recurrences^5^ and incomplete recovery.^14^ Analytical approaches such as latent class analysis (LCA) have come to the forefront to capture the complex transitions typical of LBP trajectories, focusing on individual pain reports rather than broad time-based categories.^2,7,8,20^

Emerging research using LCA to identify individual trajectories in acute LBP has begun highlighting the diversity of trajectories and the influence of different biopsychosocial factors at baseline. Downie et al.^6^ evaluated 1585 patients over 12 weeks and found 5 pain trajectory groups. These were rapid recovery by week 2 (35.8%), recovery by week 12 (34.3%), incomplete recovery (14.0%), fluctuating pain (10.5%), and persistent high pain (5.4%). Factors such as higher pain intensity and workers' compensation correlated with the persistent high-pain group, and beliefs about pain persistence were associated with nonrecovery. Schuller et al.,^31^ who studied 1377 LBP patients with mixed pain durations over 6 months, identified 3 trajectories: persistent high pain (n = 226), persistent pain with substantial improvement (n = 578), and mild pain with moderate improvement (n = 313). Baseline factors such as male gender and previous specialist consultations had limited predictive value. Da Silva et al.^32^ studied 542 older adults over 12 months, distinguishing 3 trajectories: pain recovery (n = 31), incomplete recovery (n = 253), and persistent severe pain (n = 258). Factors such as low education and depressive symptoms correlated with the persistent severe LBP trajectory.

However, there is a paucity of inception cohort studies investigating the trajectories from the onset of a new episode of acute LBP over one year. To address this knowledge gap, the primary objective of our study was to conduct a community-based inception cohort study to identify distinct individual trajectories of participants with a new episode of acute LBP over one year, including participants who do not necessarily seek professional health care because of their acute LBP. Our secondary objective was to quantify the association of biopsychosocial variables at baseline with trajectory class membership.

## 2. Methods

### 2.1. Study design and setting

We conducted a longitudinal, community-based, inception cohort study to evaluate the course of acute LBP over 52 weeks. Low back pain was defined as pain between the lower edge of the 12th rib and the gluteal folds, with or without radiating pain to one or both legs.^16^ The study was conducted at 5 participating sites. Baseline data were collected within 4 weeks of LBP onset. Follow-up data were obtained 8, 12, 26, and 52 weeks after LBP onset. All participants gave informed consent before their first examination. The study adhered to the tenets of the Declaration of Helsinki and was approved by the local ethics committee (BASEC-No. 2016-02096). We followed the guidelines for reporting on latent trajectory studies (GRoLTS) for data analysis and reporting^35^ and a recently published framework for constructing and interpreting latent class trajectory models.^24^

### 2.2. Recruitment and eligibility criteria

We recruited participants through advertisements (newspapers, flyers, mailing lists, word of mouth, local university websites, and nearby local hospitals and private practices). Participants were eligible if they (1) had acute LBP with or without leg pain of less than 4 weeks duration (in the case of a recurrent episode, participants had to be pain-free for at least 3 months before the acute pain onset), (2) were between 18 and 65 years of age, (3) had Internet access, and (4) were able to read and understand German. Participants were excluded if they met any of the following criteria: evidence of severe pathology (LBP because of trauma, tumor, infection, anomaly, or evidence of cauda equina syndrome), history of LBP surgery, current psychological problems requiring psychological treatment, and current pregnancy or first year postpartum. Eligibility criteria were determined by telephone or e-mail, and reasons for exclusion were not recorded. Investigators reconfirmed eligibility criteria before the first clinical evaluation.

### 2.3. Data collection

Data collection included clinical assessments and online surveys, although the results of the clinical assessments are not reported in this analysis. Pain intensity as primary outcome was derived at baseline and follow-up time points from the mean of 3 self-reported 11-point numeric rating scales (NRS) for current, worst, and average pain in the past week. Online surveys included sociodemographic data (age, sex, education level, work status, and quality of life). In addition, we collected LBP-related factors on pain frequency, previous LBP episodes, referred pain, pain localization, treatment, medication, imaging, and disability (Oswestry disability index [ODI]).^26^ Psychological factors such as illness perception (illness perception questionnaire [IPQ]^27^), avoidance/endurance behavior (avoidance endurance questionnaire [AEQ]^12^), pain vigilance (pain vigilance awareness questionnaire [PVAQ]^23^), depression and stress (Depression Stress Anxiety Scale [DASS]^28^), anxiety (State-Trait Anxiety Inventory [STAI]^33^), and risk of developing persistent pain (Keele Start Back Screening Tool [STarT])^17^ were collected. A detailed description of the measures and a data collection schedule can be found in Supplementary files 1 (Table S1, http://links.lww.com/PR9/A228) and 2 (Table S2, http://links.lww.com/PR9/A228). Dropout was defined as missing data at 2 consecutive time points or a participant's withdrawal from the study, with reasons for withdrawal ascertained.

### 2.4. Statistical analysis

Descriptive statistics characterized the sample at baseline. The statistical analysis included 2 phases to meet the study's objectives. A sensitivity analysis was conducted to compare baseline characteristics between participants who were retained and those who dropped out.

### 2.5. Phase 1: latent class linear mixed model

We used a latent class linear mixed model (LCMM) to identify homogeneous latent class pain trajectories over time. Latent class linear mixed model extends linear mixed models and latent class growth analysis by accounting for individual variability and latent group structure.^29^ The time metric was measured in weeks since the onset of acute LBP (according to follow-up time points). Pain intensity as a dependent variable was measured on a continuous scale. The LCMM assumes that data are missing at random (MAR) using maximum likelihood estimation.

First, we used spaghetti plots to inspect individual pain trajectories over time visually. We then applied a stepwise procedure assuming one underlying latent class (G = 1) in the study population. We then tested additional models by increasing the latent classes (G > 1–5) to derive the best model fit for the data. We included linear, quadratic, and cubic time as fixed-effects terms in all our models (G 1–5) to account for nonlinear growth trajectories over time. The LCMM approach capitalized on its inherent flexibility to use all available data, under the MAR assumption. This enabled the inclusion of every participant's data, up to their last available measurement, ensuring comprehensive utilization of the data set despite varying levels of completeness. In addition, the model accounted for random effects associated with time, acknowledging that although there is an overarching pattern of pain over time, individual participants might exhibit unique trajectories. Furthermore, the mixture component of the model, which also incorporated linear, quadratic, and cubic time terms, was employed to detect latent subgroups within the data, identifying distinct pain trajectories over time for these subgroups. An automatic grid search function was performed with a maximum of 30 iterations from 100 random vectors of initial values to avoid converging to local maxima.^15^ We used Akaike information criterion (AIC),^1^ the Bayesian information criterion (BIC),^10^ the size-adjusted Bayesian information criterion (SABIC), and the Lo-Mendell-Rubin likelihood ratio test (LMR-LRT)^25^ as goodness-of-fit criteria to assess model fit and identify the optimal number of latent classes (G = 1–5). Goodness-of-fit criteria are based on log-likelihood and several parameters to account for model complexity. Lower BIC, AIC, and SABIC values and significant LMR-LRT tests indicate better model fit. After model selection, the average posterior probability assignment (APPA), the odds of correct classification (OCC), and relative entropy were used to assess model adequacy, with APPA greater than 0.7, OCC greater than 5.0, and relative entropy greater than 0.5 considered acceptable.^24^ Other criteria were a minimum class proportion of 5% and clinical relevance of latent class trajectories. The R code used for the LCMM and model comparisons is provided in Supplementary Material S4, http://links.lww.com/PR9/A228.

### 2.6. Phase 2: multinomial logistic regression analysis

Multinomial logistic regression analysis was used to model the latent class membership as a linear function of a priori selected biopsychosocial factors at baseline, whereas the most favorable class served as the reference category. An OR >1 means that a one-unit increase in the independent variable is associated with an increased chance of being assigned to the current class compared with the reference class. We tested for multicollinearity by calculating correlations between independent variables. Categorical independent variables were dichotomized (see Supplementary Material S1, Table S1, http://links.lww.com/PR9/A228).

First, univariable multinomial logistic regression analysis was performed to calculate unadjusted ORs with 95% confidence intervals (95% CI) to identify significant associations of independent variables with latent class membership. Second, multivariable multinomial regression analysis was performed to calculate adjusted ORs with 95% CI for the effects of the remaining significant variables. We used a penalized version of multivariable analysis to account for perfect separation problems. All analyses were performed with the statistical software R (v. 4.0.5, packages “lcmm,”^29^ “LCTMtools,”^24^ “tidyLPA,”^30^ “nnet,”^37^ “arsenal,”^13^ “brglm2”^21^ and “detectseparation”^22^).

## 3. Results

**Figure 1.**
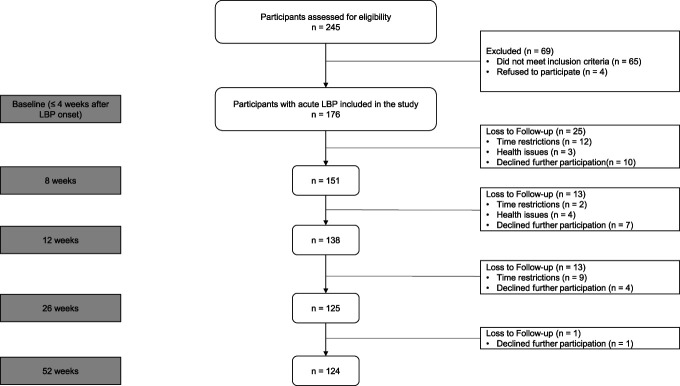
Flowchart of participants through the study.

Figure 1 shows the study flow chart. A total of 176 participants (mean age 39.1 years, 50.9% female) with acute LBP were enrolled between November 2017 and February 2021. Overall, cumulative loss to follow-up was 25 (14.2%), 38 (21.6%), 51 (28.9%), and 52 (29.5%) at 8, 12, 26, and 52 weeks, respectively. In our analysis, 141 of the total 176 participants had at least 3 follow-up measurements, providing a robust data set for the LCMM to effectively capture the trajectories over time. Table 1 shows sample characteristics at baseline.

**Table 1:** Baseline sample characteristics for each class of the final 4-class model.

	All participants (n = 176)	Class 1 = mild/moderate fluctuating pain (n = 95)	Class 2 = delayed recovery by week 52 (n = 11)	Class 3 = persistent moderate pain (n = 58)	Class 4 = moderate/severe fluctuating pain (n = 12)	*P*
Sociodemographic variables						
Age (y)*	39.3 (13.2)	39.7 (13.2)	41.1 (13.9)	36.6 (12.5)	47.5 (12.9)	0.059
Female (%)*	89 (50.9)	42 (44.2)	7 (63.6)	30 (52.6)	10 (83.3)	0.057
Lower educational level (%)*	66 (38.6)	31 (33.7)	5 (45.5)	24 (42.1)	6 (54.5)	0.453
No work (%)	12 (6.8)	7 (7.4)	0 (0.0)	4 (6.9)	1 (8.3)	0.827
≤Moderate QoL (%)*	72 (40.9)	34 (35.8)	5 (45.5)	25 (43.1)	8 (66.7)	0.209
LBP-related variables						
Pain (NRS)*	4.6 (1.6)	4.1 (1.4)	5.3 (1.5)	4.7 (1.0)	7.1 (1.5)	**<0.001**
Daily frequent pain (%)*	117 (67.2)	60 (63.2)	7 (70.0)	41 (71.9)	9 (75.0)	0.648
≥1 previous LBP episode (%)*	134 (77.0)	66 (69.5)	7 (70.0)	52 (91.2)	9 (75.0)	**0.020**
Referred pain (%)	85 (48.9)	39 (41.1)	8 (80.0)	31 (54.4)	7 (48.9)	0.062
Most disturbing pain region (%)†						0.290
Back	154 (88.5)	82 (86.3)	7 (70.0)	54 (94.7)	11 (91.7)	
Legs	12 (6.9)	9 (9.5)	1 (10.0)	1 (1.8)	1 (8.3)	
Pins/needles	5 (2.9)	3 (3.2)	1 (10.0)	1 (2.1)	0 (0.0)	
Other	3 (1.7)	1 (1.1)	1 (10.0)	1 (1.8)	0 (0.0)	
Treatment due to LBP (%)*‡	108 (63.9)	54 (58.1)	8 (80.0)	36 (66.7)	10 (83.3)	0.200
LBP medication (%)*†	77 (44.3)	35 (36.8)	7 (70.0)	28 (49.1)	7 (58.3)	0.097
Imaging (%)	34 (19.3)	19 (20.0)	3 (27.3)	9 (15.5)	3 (25.0)	0.738
Disability (ODI)*	17.0 (10.5)	14.5 (9.4)	19.3 (12.3)	17.4 (8.8)	32.2 (12.5)	**<0.001**
Psychological variables						
Illness perception: timeline (IPQ)*	2.3 (1.0)	2.2 (1.0)	2.4 (1.2)	2.5 (1.0)	2.5 (0.6)	0.444
Illness perception: control (IPQ)*	1.8 (0.6)	1.7 (0.6)	1.9 (0.5)	1.7 (0.6)	2.0 (0.7)	0.288
Illness perception: reason for pain (%)§						
Bodily causes	156 (88.6)	87 (91.5)	7 (63.6)	52 (89.6)	10 (83.3)	**<0.001**
Psychological causes	47 (26.7)	22 (23.2)	3 (27.2)	20 (34.5)	2 (16.7)	**<0.001**
Other causes	25 (14.2)	13 (13.7)	3 (27.2)	8 (13.8)	1 (8.3)	**0.003**
Endurance behavior (AEQ)*	2.6 (1.1)	2.5 (1.2)	2.3 (1.1)	2.8 (1.0)	2.3 (1.4)	0.176
Avoidance behavior (AEQ)*	3.4 (1.6)	3.5 (1.6)	3.9 (1.8)	3.3 (1.5)	2.6 (1.8)	0.219
Pain vigilance (PVAQ)*	36.9 (11.3)	36.8 (11.2)	38.5 (13.9)	36.4 (10.6)	38.9 (13.7)	0.873
Depression (DASS21)*	5.3 (6.4)	5.1 (6.2)	5.3 (5.5)	4.7 (5.9)	10.2 (9.8)	0.089
Stress (DASS21)*	9.0 (7.0)	8.2 (6.5)	11.6 (7.2)	9.0 (7.2)	11.6 (10.4)	0.278
Anxiety (STAI-S)*	37.6 (10.2)	36.8 (10.4)	39.5 (7.7)	37.5 (9.4)	42.6 (13.9)	0.356
Risk stratification (%, SBT)*						**<0.001**
Low	117 (69.6)	68 (74.7)	6 (60.0)	41 (73.2)	2 (18.2)	
Medium	42 (25.0)	20 (22.0)	2 (20.0)	14 (25.0)	6 (54.5)	
High	9 (5.4)	3 (3.3)	2 (20.0)	1 (1.8)	3 (27.3)	

Numbers are means (standard deviations) of participants unless stated otherwise.

Entries in bold indicate statistically significant *P*-values (*P* < 0.05).

* Variables included in the multinomial logistic regression model.

† Categorical data do not sum up to 100% due to missing values.

‡ Treatment includes either general practitioner, specialist, pain specialist, psychologist, physiotherapy, massage, or chiropractic consultations due to LBP.

§ Multiple answers possible.

AEQ, avoidance endurance questionnaire; DASS21, Depression Anxiety Stress Scale; IPQ, illness perception questionnaire; LBP, low back pain; NRS, numeric rating scale; ODI, Oswestry disability index; PVAQ, pain vigilance and awareness questionnaire; QoL, quality of life; SBT, Start Back Tool; STAI-S, State Trait Anxiety Inventory—State.

### 3.1. Latent class linear mixed model analysis: acute low back pain trajectories

After visual inspection of the data (see Supplementary Material 3, Figure S3, http://links.lww.com/PR9/A228) and a significant decrease in AIC and BIC when using cubic time effects in the models, linear and quadratic models were discarded from further analyses. Details of the discarded models are provided in Supplementary Material 3 (Table S3, http://links.lww.com/PR9/A228).

Table 2 shows all models' goodness-of-fit criteria and class proportions (G = 1–5). The 5-class model had the lowest AIC, whereas the 2-class and 4-class models had the lowest BIC values. When sample size was considered, the 5-class model had the lowest SABIC. Relative entropy was adequate for all models, with 0.67, 0.72, and 0.74, respectively. Class proportions were above 5% in all models, whereas the 5-class model had 3 classes with few participants compared with only 2 classes with few participants in the 4-class model. Lo-Mendell-Rubin Likelihood Ratio Test showed a significant improvement in model fit for one additional class for each model included.

**Table 2:** Goodness-of-fit criteria and class proportions for 1-class to 5-class models.

Goodness-of-fit criteria						LMR-LRT (*P*)	Proportions per class %				
Model	G	AIC	BIC	SABIC	Relative entropy		Class 1	Class 2	Class 3	Class 4	Class 5
M1	1	2774.9	2809.8	2774.6	1.0	—	100				
M2	2	2739.4	2790.2	2739.5	0.67	*P* < 0.001	64.8	35.2			
M3	3	2729.5	2796.1	2729.6	0.72	*P* < 0.002	63.6	6.3	30.1		
M4	4	2707.9	2790.3	2707.9	0.74	*P* < 0.001	54.0	6.2	33.0	6.8	
M5	5	2702.7	2801.0	2702.9	0.74	*P* = 0.014	43.2	8.0	7.4	35.2	6.2

Model M1-5 using cubic fixed effects for up to 5 classes.

AIC, akaike information criterion; BIC, Bayesian information criterion; G, number of classes; LMR-LRT, Lo-Mendell-Rubin likelihood ratio test (*P* values <0.05 indicating statistically significant improvement of model fit for one additional class); SABIC, size-adjusted Bayesian information criterion.

Table 3 shows additional model selection tools. Average posterior probability assignment ranged from 0.79 to 0.91, whereas in the 4-class model, all values were equal to or greater than 0.80. The OCC of all included models was greater than 5. Considering the similarity of the AIC values between the 4-class and 5-class models, the lower BIC value of the 4-class model, adequate additional model selection tools, and clinical relevance, the 4-class model was chosen to best represent acute LBP trajectories over one year in our data.

**Table 3:** Additional model selection tools.

Number of classes	Model 2		Model 3		Model 4		Model 5	
	APPA	OCC	APPA	OCC	APPA	OCC	APPA	OCC
Class 1	0.91	5.86	0.89	5.38	0.89	7.44	0.81	10.38
Class 2	0.85	10.45	0.82	51.48	0.81	45.38	0.80	34.17
Class 3			0.79	9.13	0.80	8.76	0.81	40.20
Class 4					0.80	51.25	0.79	8.42
Class 5							0.86	84.56

Model 1 not included in the table.

APPA, average posterior probability assignment (overall average probability of assignment to each class, should be >0.7 for each class); OCC, odds of correct classification (ratio of the odds of a correct classification into each class, should be >5.0).

**Figure 2.**
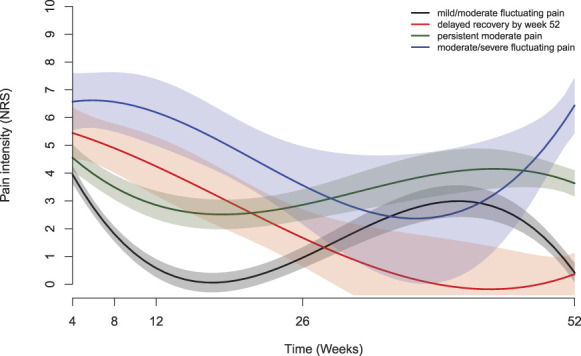
Class-specific mean predicted acute LBP trajectories. LBP, low back pain.

**Figure 3.**
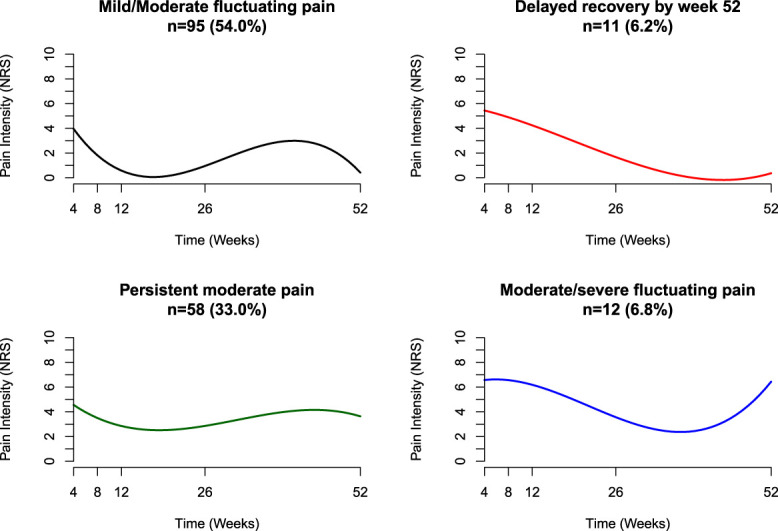
Detailed description of each class.

Figures 2 and 3 illustrate the mean predicted trajectories of the final 4-class model and the detailed characteristics of each trajectory of acute LBP. Of the participants, 54.0% (n = 95) followed the “mild/moderate fluctuating pain” trajectory, which showed an initial rapid decrease in mean pain intensity from a baseline of NRS 4.1/10, followed by a pain increase at subsequent measurement points; 6.2% (n = 11) fit the “delayed recovery by week 52” trajectory, starting with a baseline mean pain intensity of NRS 5.3/10 and experiencing a gradual decrease in pain intensity over time. 33.0% (n = 58) matched the “persistent moderate pain” trajectory, maintaining a consistent moderate pain level after an initial mean intensity of NRS 5.3/10. Finally, 6.8% (n = 12) aligned with the “moderate/severe fluctuating pain” trajectory, beginning with a baseline intensity of NRS 7.1/10, with a reduced pain intensity by week 26 but then seeing an increase by week 52.

### 3.2. Comparison of baseline characteristics within classes

Pain intensity at baseline differed between classes (*P* < 0.001), with higher pain intensities reported in less favorable trajectories. More participants in less favorable classes reported having at least one previous episode of LBP (*P* = 0.020), whereas 23% never had an episode of LBP. Participants assigned to less favorable classes reported more significant disability (*P* < 0.001) and significant differences were observed in baseline risk stratification for developing persistent pain, as assessed by the SBT, across the different trajectory classes (*P* < 0.001). There were significant differences between illness perception categories regarding the reasons for pain (*P* < 0.001).

### 3.3. Sensitivity analysis

Our sensitivity analysis (see Supplementary Material S4, Table S4, http://links.lww.com/PR9/A228) highlighted significant differences between participants who completed the study and dropouts in disability (ODI) scores, work status, and avoidance/endurance behavior (AEQ).

### 3.4. Multinomial logistic regression analysis

The most favorable latent class, “mild/moderate fluctuating pain,” was a reference class. After testing for collinearity between independent variables, the variable stress was removed from further analyses. Table 4 shows the univariable (unadjusted) and multivariable (adjusted) multinomial logistic regression analysis results.

**Table 4:** Multinomial logistic regression models.

Baseline predictors	Unadjusted OR (95% CI)	Adjusted OR (95% CI)
Sociodemographic variables		
Age		
Mild/moderate fluctuating pain	1.0 (reference)	
Delayed recovery by week 52	1.01 (0.96–1.06)	
Persistent moderate pain	0.99 (0.96–1.01)	
Moderate/severe fluctuating pain	1.05 (1.00–1.10)	
Sex (female)		
Mild/moderate fluctuating pain	1.0 (reference)	1.0 (reference)
Delayed recovery by week 52	2.21 (0.60–8.05)	1.91 (0.53–6.85)
Persistent moderate pain	1.40 (0.72–2.71)	1.81 (0.85–3.82)
Moderate/severe fluctuating pain	6.31 (1.31–30.37)	5.07 (0.95–27.16)
Educational level (low)		
Mild/moderate fluctuating pain	1.0 (reference)	
Delayed recovery by week 52	1.64 (0.46–5.80)	
Persistent moderate pain	1.43 (0.72–2.82)	
Moderate/severe fluctuating pain	2.36 (0.67–8.35)	
QoL (≤moderate)		
Mild/moderate fluctuating pain	1.0 (reference)	
Delayed recovery by week 52	1.49 (0.42–5.26)	
Persistent moderate pain	1.36 (0.69–2.65)	
Moderate/severe fluctuating pain	3.56 (1.00–12.80)	
Pain-related variables		
Pain at baseline (NRS)		
Mild/moderate fluctuating pain	1.0 (reference)	1.0 (reference)
Delayed recovery by week 52	1.99 (1.22–3.25)	1.77 (0.99–3.18)
Persistent moderate pain	1.47 (1.13–1.91)	1.53 (1.10–2.15)
Moderate/severe fluctuating pain	4.91 (2.68–9.01)	2.87 (1.35–6.13)
Pain frequency (daily)		
Mild/moderate fluctuating pain	1.0 (reference)	
Delayed recovery by week 52	1.36 (0.33–5.60)	
Persistent moderate pain	1.49 (0.73–3.04)	
Moderate/severe fluctuating pain	1.74 (0.44–6.89)	
Previous LBP episodes (≥1)		
Mild/moderate fluctuating pain	1.0 (reference)	1.0 (reference)
Delayed recovery by week 52	1.02 (0.24–4.24)	2.07 (0.47–9.11)
Persistent moderate pain	4.56 (1.65–12.62)	8.02 (2.45–26.21)
Moderate/severe fluctuating pain	1.31 (0.33–5.23)	6.84 (1.06–44.27)
Treatment (yes)		
Mild/moderate fluctuating pain	1.0 (reference)	
Delayed recovery by week 52	2.88 (0.58–14.35)	
Persistent moderate pain	1.44 (0.71–2.90)	
Moderate/severe fluctuating pain	3.61 (0.74–17.41)	
LBP medication (yes)		
Mild/moderate fluctuating pain	1.0 (reference)	
Delayed recovery by week 52	4.00 (0.97–16.47)	
Persistent moderate pain	1.65 (0.85–3.22)	
Moderate/severe fluctuating pain	2.40 (0.70–8.13)	
Disability (ODI)		
Mild/moderate fluctuating pain	1.0 (reference)	1.0 (reference)
Delayed recovery by week 52	1.05 (0.99–1.13)	1.03 (0.95–1.11)
Persistent moderate pain	1.04 (1.01–1.08)	1.03 (0.97–1.08)
Moderate/severe fluctuating pain	1.15 (1.08–1.22)	1.05 (0.94–1.16)
Psychological variables		
Illness perception: timeline (IPQ)		
Mild/moderate fluctuating pain	1.0 (reference)	
Delayed recovery by week 52	1.26 (0.65–2.43)	
Persistent moderate pain	1.29 (0.92–1.80)	
Moderate/severe fluctuating pain	1.32 (0.71–2.48)	
Illness perception: control (IPQ)		
Mild/moderate fluctuating pain	1.0 (reference)	
Delayed recovery by week 52	1.58 (0.54–4.58)	
Persistent moderate pain	0.83 (0.48–1.43)	
Moderate/severe fluctuating pain	2.02 (0.76–5.33)	
Endurance behavior (AEQ)		
Mild/moderate fluctuating pain	1.0 (reference)	
Delayed recovery by week 52	0.88 (0.51–1.51)	
Persistent moderate pain	1.34 (0.98–1.83)	
Moderate/severe fluctuating pain	0.86 (0.51–1.44)	
Avoidance behavior (AEQ)		
Mild/moderate fluctuating pain	1.0 (reference)	
Delayed recovery by week 52	1.18 (0.77–1.82)	
Persistent moderate pain	0.94 (0.76–1.15)	
Moderate/severe fluctuating pain	0.71 (0.49–1.03)	
Pain vigilance (PVAQ)		
Mild/moderate fluctuating pain	1.0 (reference)	
Delayed recovery by week 52	1.01 (0.95–1.07)	
Persistent moderate pain	0.99 (0.96–1.02)	
Moderate/severe fluctuating pain	1.01 (0.95–1.07)	
Depression (DASS)		
Mild/moderate fluctuating pain	1.0 (reference)	
Delayed recovery by week 52	1.01 (0.91–1.11)	
Persistent moderate pain	0.98 (0.93–1.05)	
Moderate/severe fluctuating pain	1.08 (1.00–1.17)	
Anxiety (STAI-S)		
Mild/moderate fluctuating pain	1.0 (reference)	
Delayed recovery by week 52	1.02 (0.96–1.09)	
Persistent moderate pain	1.01 (0.97–1.04)	
Moderate/severe fluctuating pain	1.05 (0.99–1.12)	
Risk stratification (SBT, ≥ medium category)		
Mild/moderate fluctuating pain	1.0 (reference)	1.0 (reference)
Delayed recovery by week 52	1.97 (0.51–7.60)	1.14 (0.26–4.98)
Persistent moderate pain	1.08 (0.50–2.30)	0.77 (0.30–1.93)
Moderate/severe fluctuating pain	13.30 (2.67–66.13)	2.99 (0.46–19.39)

Unadjusted and adjusted multinomial logistic regression analysis to model the OR of membership in each latent class as linear function of biopsychosocial factors at baseline. Mild/moderate fluctuating pain class serves as reference category.

95%, 95% confidence interval; AEQ, avoidance endurance questionnaire; DASS, depression anxiety stress scale; LBP, low back pain; NRS, numeric rating scale; ODI, Oswestry disability index; OR, odds ratio; PVAQ, pain vigilance awareness questionnaire; QoL, quality of life; SBT, Start Back Tool; STAI-S, State Trait Anxiety Inventory—State.

Women were more likely to be classified as having “moderate/severe fluctuating pain” than “mild/moderate fluctuating pain” in the unadjusted model, but this association was not significant in the adjusted model. Participants with increased baseline pain intensity were more likely to be classified as having a “delayed recovery by week 52,” “persistent moderate pain,” and “moderate/severe fluctuating pain” than “mild/moderate fluctuating pain” in the unadjusted analysis. The adjusted analysis maintained this association with both “persistent moderate pain” and “moderate/severe fluctuating pain” trajectories.

Participants with a history of previous LBP episodes were more likely to be classified into the “persistent moderate pain” class in both the unadjusted and adjusted models. In addition, the adjusted model revealed increased odds of having “moderate/severe fluctuating pain” in comparison to “mild/moderate fluctuating pain” for participants with at least one previous LBP episode. Although participants with increased disability at baseline were more likely to have “persistent moderate pain” and “moderate/severe fluctuating pain” in the unadjusted analysis, these associations did not remain significant in the adjusted model.

Psychological variables measured at baseline did not yield significant associations with less favorable pain classes. In the unadjusted analysis, participants with a medium or high risk of persistent future disabling LBP, as classified by the Start Back Tool, were more likely to experience “moderate/severe fluctuating pain.” This association did not remain significant in the adjusted model.

## 4. Discussion

In our community-based inception cohort study, we identified 4 distinct trajectories representing the course of acute LBP over one year, including participants who did not seek professional health care because of their acute LBP. Our results indicate that the course of acute LBP was not as favorable as previously reported,^4^ with a significant proportion of participants (46%) experiencing less favorable trajectories. Even without accounting for clinical assessments in our study, these results highlight the complexity and heterogeneity of the course of acute LBP and the need for better understanding of the factors contributing to adverse outcomes.

The unadjusted analysis revealed associations between less favorable trajectories and several baseline factors, including being female, pain intensity, previous episodes of LBP, disability, and being classified as having a medium or high risk of persistent LBP. Unexpectedly, psychological variables did not reveal significant associations with less favorable trajectories. However, in the adjusted model, only pain intensity at baseline and previous episodes of LBP remained significantly associated with less favorable trajectories.

### 4.1. Trajectories of acute low back pain

Our study, the first to delineate acute LBP trajectories over a year in a community-based sample, included participants not seeking professional health care for their symptoms. Although we identified a single favorable trajectory (mild/moderate fluctuating pain) covering 54.0% of the sample, Downie et al.^6^ found 5 trajectories in a sample of care-seeking participants, with 3 indicating rapid pain recovery (35.8%), incomplete recovery (14.0%), and recovery by week 12 (34.3%), respectively. This last trajectory is similar to our “delayed recovery to week 52” but with fewer participants at 6.2% (n = 11). The difference may be because of their 12-week follow-up of acute LBP vs our up-to-52-week follow-up.

In our cohort, only 63.9% of the participants sought care for acute LBP compared with 100% in the study by Downie et al. This may explain the higher baseline pain intensity (6.3 vs 4.6) and self-reported disability (54.3% vs 17.0%). Although our study only identified baseline pain intensity and history of LBP episodes as common factors, the trajectories of a cohort seeking care for their acute LBP may explain the association of factors such as increased perceived risk of persistence, longer duration of acute LBP, and poor quality of life with nonfavorable trajectories in the study by Downie et al.

Da Silva et al.,^32^ focusing on older participants (>55 years) with acute LBP being referred to public and private healthcare professionals, identified 3 distinct acute LBP trajectories. Notably, only 6% of their participants were classified into a pain recovery trajectory, whereas the remainder were almost evenly split between incomplete pain recovery (46%) and persistent severe pain (48%) trajectories. Our study's demographics differed substantially, with a mean age of 39.3 years against their 68 years and a gender distribution of 50.9% females compared with 86%. In addition, only 44.3% took medication for their acute LBP in our study in comparison to 74% in the study of da Silva. Moreover, their report of elevated baseline pain intensities and disability could further elucidate the higher percentage (47.6%) assigned to their persistent severe pain trajectory, as opposed to the 33.0% and 6.8% in our persistent moderate pain and moderate/severe fluctuating pain trajectories, respectively. These different sociodemographic and clinical data could partially account for the higher proportion of participants in nonfavorable pain trajectories in the study by da Silva et al.

Although our analysis identified 4 distinct trajectories of acute LBP over one year, Kongsted et al.^20^ delineated up to 12 pain trajectories using more frequent, weekly measurements of pain intensity. Their study is consistent with our study not only in identifying similar patterns ranging from mild (episodic) to severe (ongoing) trajectories but also extends to include a recovery trajectory and other progressively improving patterns. It is noteworthy that in the study by Kongsted et al., approximately 71% of patients reported pain duration of 4 weeks or less, compared with our cohort where all participants were within this acute LBP time frame. The larger sample size (n = 1082) and more frequent data collection in their study likely contributed to the identification of a broader range of trajectories. Despite these methodological differences, the consistency in identifying similar trajectories between the 2 studies underscores the validity of using nonlinear models to understand LBP progression. This comparison highlights that although more frequent data collection, as in the study by Kongsted et al., can capture greater variability and potentially reveal more detailed trajectories, significant and meaningful patterns can still be identified with less frequent measurements, as demonstrated in our study. The key implication is that most LBP do not follow a straightforward path of recovery or chronicity, and both frequent and less frequent data collection approaches contribute valuable insights into the complex nature of LBP trajectories.

### 4.2. Strengths and limitations of the study

Our investigation into acute LBP trajectories has several notable strengths and limitations. On the strength side, our study contributes a novel perspective because it is the first to document a person-centered approach by delineating trajectories of participants with acute LBP over one year, including participants who do not seek professional health care because of their acute LBP. We further employed robust statistical methodologies, enhancing existing latent class analysis approaches, to account for intraindividual and interindividual variability of self-perceived pain intensities over time. Including an extensive array of baseline biopsychosocial factors furnishes a comprehensive insight into acute LBP's complexity in a community-based sample and its influence on the identified 4 distinct trajectories.

Conversely, our study has some limitations. First, we observed a high loss to follow-up rate of 29.3% at the 52-week mark, which is common in observational studies. The study measures and follow-up procedures resulted in an extensive burden and may explain the reported attrition. The sensitivity analysis suggests potential limitations in the generalizability of the findings related to disability and behavioral outcomes across different populations.

Compared with Swiss Federal Statistical Office data,^34^ our sample had a lower proportion of participants with below tertiary education (38.6% vs 55.3%), possibly influencing the low averaged disability and maladaptive behaviors reported. In addition, our inclusion of participants unable to work because of LBP resulted in a slight overrepresentation of no work status (6.8% vs 4.0% community data), which may affect the extrapolation of employment-related outcomes.

The complexity of the latent class linear mixed model, combined with the small proportions observed in certain classes, necessitates a cautious interpretation of the results. For instance, one of the identified classes included only 11 participants, which may impact the robustness and generalizability of our findings. Furthermore, our study's reliance on just 5 follow-up time points might limit our ability to capture more nuanced changes in pain trajectories over time, eg, recovery or fluctuating patterns. Although biopsychosocial variables were measured at all time points, our multinomial logistic regression model only included baseline values. Using time-varying data for independent variables could provide a more accurate explanation of acute LBP trajectories over time.

### 4.3. Clinical implications and future research agenda

The results of our study challenge the common but outdated notion that acute LBP is uniformly favourable. This finding is critical given the increasing recognition of LBP as a long-term condition with the potential for recurrent episodes. Consequently, patients with acute LBP must know this potential for future episodes.

Future research should consider the potential for improved health outcomes by subgrouping based on LBP trajectories in clinical trials. This proposed approach may be key to more effective, tailored treatment interventions for LBP. Future research may also focus on the likelihood of transitions between distinct trajectories of acute LBP over time. This would further enhance the understanding of targeted treatment interventions.

## Disclosures

The authors have no conflicts of interest to declare. This work was supported by the Swiss National Science Foundation (Grant No.173297).

## Supplemental digital content

Supplemental digital content associated with this article can be found online at http://links.lww.com/PR9/A228.

## Supplementary Material

SUPPLEMENTARY MATERIAL
