# Supplementary material for: The course of acute low back pain: a community-based inception cohort study
Source: Pain Rep. 2024 Apr 10;9(3):e1152. doi: 10.1097/PR9.0000000000001152 (PMC11008624; doi:10.1097/PR9.0000000000001152)
Supplement: SUPPLEMENTARY MATERIAL [file painreports-9-e1152-s001.pdf]

The course of acute Low Back Pain: a community-based inception cohort study

Supplementary Materials

Supplementary Material 1 (S1)

Table S1: Description of measures (survey data)

**Measure**	**Description**	**Units/data type/range + Coding of data in regression model**
Demographic		
Age	Self-reported year of birth	Years
Sex	Self-reported sex	Categorical
Social		
Educational level	Self-selected highest level of education (no completed vocational training, secondary level education, tertiary level education) from a list	Categorical<br>Coding: lower educational level (no completed vocational training, secondary level education) vs. higher educational level (tertiary education)
Work status	Self-selected (no work due to LBP, unemployed, vocational training, part-time, full-time) from a list	Not included in the regression model
Quality of life	Self-selected (very poor, poor, moderate, good, very good) from a list	Categorical<br>Coding: ≤ moderate vs. > moderate
Details of Low Back Pain		

Pain intensity (NRS)	Self-reported pain intensity (numeric rating scale): average score of 1) current pain intensity, 2) mean pain intensity the last week and 3) maximum pain intensity the last week	0-10: higher scores indicating more pain
Pain frequency	Self-reported pain frequency (several times per day, once a day, several times per week, once a week, monthly, occasionally) from a list	Categorical<br>Coding: daily frequent pain (several times a day, once a day) vs. weekly or occasional (several times per week, once a week, monthly, occasionally)
Previous episodes	Self-selected previous LBP episodes (never, 1-2 episodes, 3-4 episodes, ≥4 episodes) from a list	Categorical<br>Coding: ≥ one previous episode vs. no previous episodes
Referred pain	Self-reported referred pain (yes/no) into other body regions	Not included in the regression model
Most disturbing pain region (localization)	Self-selected (Back, Legs, Pins/Needles or Other) from a list.	Not included in the regression model
Treatment due to LBP	Self-reported type of treatment (no, General Practitioner, Specialist, Pain Specialist, Psychologist, Physiotherapy, Chiropractor, Massage)	Categorical<br>Coding: yes (any kind of treatment)/no
Medication	Self-selected medication due to LBP (yes/no): analgesics, opioids, antidepressants, muscle relaxants, cannabis-related products, other) from a list	Categorical<br>Coding: yes (any kind of medication)/no
Imaging	Self-selected type of imaging due to LBP (no, X-Ray, MRI, CT, other)	Not included in the regression model
Disability (ODI)	Questionnaire: assesses limitations of 10 different activities of daily living due to LBP. Each activity is scored	0-100: higher scores indicating greater disability

	on a 0-5 scale, with 5 representing greatest disability. The score is calculated from the sum-med score of the individual items divided by the maximal possible score of 50 multiplied by 100. One item (sex life) was removed; therefore the denominator was reduced by 5.	
Psychological		
Pain vigilance (PVAQ)	Questionnaire: assesses the habitual attention to pain. Consists of 16 items investigating the frequency of attentional habits with the focus on pain and pain change over the past 2 weeks. Each item is scaled from 0-5, whereas 2 items are inverse coded. In this study, the total score of the PVAQ was used.	0-80 (total score): higher score indicating higher overall vigilance and awareness<br>0-50 (pain score): higher scores indicating higher vigilance and awareness on pain<br>0-30 (pain change score): higher scores indicating higher vigilance and awareness on pain change
Avoidance/Endurance Behavior (AEQ)	Questionnaire: assesses self-reported fear-avoidance responses (FAR) and endurance-related responses (ER) to pain through different subscales. In this study, we used the subscale “avoidance of physical activities” as indicator of avoidance behavior and the behavioral endurance scale as an indicator of endurance behavior. Each item is scaled from 0-6.	0-6: higher score indicating more avoidance and/or endurance behavior
Depression / Stress (DASS21)	Questionnaire: assesses self-reported negative emotional states of depression, anxiety, and stress (subscales). Each subscale contains 7 items which are scaled from 0-3. Final scores are multiplied with 2.	0-42 (each subscale): higher scores indicating higher/more depression, anxiety, and stress

Anxiety (STAI-S)	Questionnaire: assesses self-perceived trait and state anxiety with 20 items for each subscale. Each item is scaled from 1-4, whereas 1 = almost never and 4 = almost always.	20-80: higher scores indicating higher state anxiety
Illness Perception (IPQ): timeline, control and causes as components of illness perception	Questionnaire: assesses individual's beliefs and feelings about their illness containing 5 components. In this study, timeline of the illness (how long participants think it will last), being in control of the illness (what participants think they can do on their own) and the perceived cause of their symptoms were investigated. Each component contains 3 items and is scaled from 1-5. For the statistical analysis of this study, the scores of the 3 items of each category were averaged.	1-5: higher scores indicating poorer prognosis and less personal control, respectively. Perceived causes of symptoms were not included in the regression model.
Start Back Tool (SBT)	Questionnaire: screens both physical and psychosocial risk factors for developing persistent low back pain. Participants are categorized into three subgroups: low, medium and high risk of persisting low back pain.	Categorical<br>Coding: low vs. medium/high risk

Numeric Rating Scale; ODI: Oswestry Disability Index; PVAQ: Pain Vigilance and Awareness Questionnaire; DASS: Depression Anxiety Stress Scale; STAI-S: State-Trait-Anxiety-Inventory – State; IPQ: Illness Perception Questionnaire; SBT: Start Back Tool

Supplementary Material 2 (S2)

Table S2: Timetable of data collection

**Measure**	**Time-point**				
	**Baseline (≤4 weeks after pain onset)**	**8 weeks**	**12 weeks**	**26 weeks**	**52 weeks**
Demographic					
Age	x				
Sex	x				
Social					
Educational level	x				
Work status	x				
Quality of life	x	x	x	x	x
Details of Lower Back Pain					
Pain intensity (NRS)	x	x	x	x	x
Pain frequency	x	x	x	x	x
Previous episodes	x	x	x	x	x
Referred pain	x	x	x	x	x
Most disturbing pain region (Localization)	x	x	x	x	x
Treatment due to LBP	x	x	x	x	x
Medication	x	x	x	x	x
Imaging	x	x	x	x	x

Disability (ODI)	x	x	x	x	x
Psychological					
Pain vigilance (PVAQ)	x	x	x	x	x
Avoidance/Endurance Behavior (AEQ)	x	x	x	x	x
Depression (DASS21)	x	x	x	x	x
Stress (DASS21)	x	x	x	x	x
State Anxiety (STAI-S)	x	x	x	x	x
Illness Perception (IPQ): timeline and control	x	x	x	x	x
Development of persisting pain					
Risk stratification Tool (SBT)	x	x	x	x	x

NRS: Numeric Rating Scale; ODI: Oswestry Disability Index; PVAQ: Pain Vigilance and Awareness Questionnaire; DASS: Depression Anxiety Stress Scale; IPQ: Illness Perception Questionnaire; SBT: Start Back Tool

Supplementary Material 3 (S3)

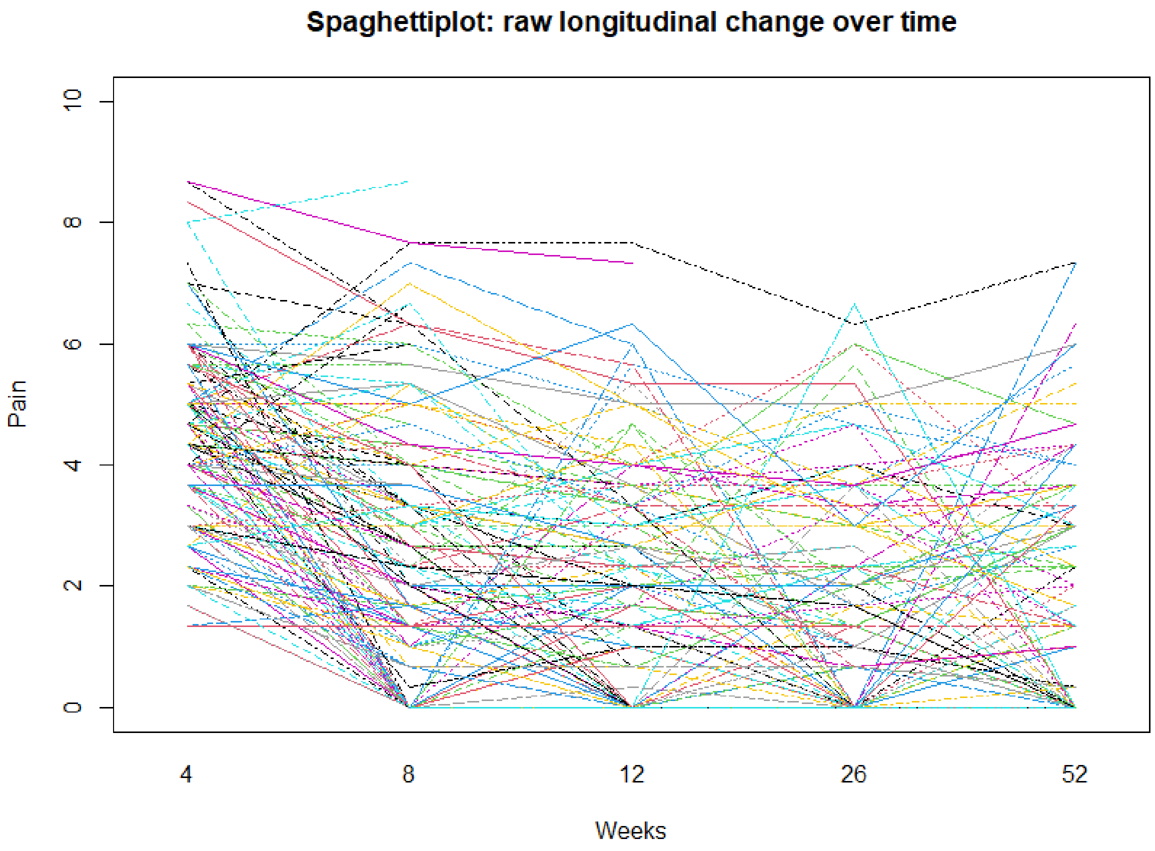

Figure S3. Raw longitudinal change of pain over time (weeks) demonstrating heterogeneity of the data.

Table S3: Detailed data of linear, quadratic and cubic models

Model	G	AIC	BIC
M1 linear	1	2965.7	2984.7
M1 quadratic	1	2856.2	2878.4
M1 cubic	1	2774.9	2809.8
M2 linear	2	2952.4	2980.9
M2 quadratic	2	2838.8	2873.7
M2 cubic	2	2739.4	2790.2
M3 linear	3	2958.4	2996.4
M3 quadratic	3	2836.9	2884.4
M3 cubic	3	2729.5	2796.1
M4 linear	4	2964.4	3011.9
M4 quadratic	4	2836.6	2896.9
M4 cubic	4	2707.9	2790.3
M5 linear	5	2970.4	3027.5
M5 quadratic	5	2838.5	2911.5
M5 cubic	5	2702.7	2801.0

Model M1-5 using linear, quadratic and cubic fixed effects for up to 5 classes; G = Number of classes; AIC = Akaike Information Criterion; BIC = Bayesian Information Criterion

Supplementary R Code for Latent Class Linear Mixed Effects Model Analysis

Fabian Pfeiffer, 22nd December 2023

Introduction

This script performs a Latent Class Linear Mixed Effects Model (LCMM) analysis to explore the relationship between pain and time, accounting for individual variability and latent group structure. The analysis was conducted using the *lcmm* package in R.

Data Description

The dataset d.fab used in this analysis consists of variables such as pain, time (time points of measurement), and ID (participant identifier). The actual data cannot be shared due to privacy concerns; however, a general structure of the dataset is provided.

Preliminary Setup

```
library(lcmm)
library(devtools)
library(tidyLPA)
```

Estimate the model with one class (G=1)

```
## Linear model
m1a<-hlme(fixed=pain~time,random=~time,subject="ID",data=d.fab)

## Quadratic model
m1b<-hlme(fixed=pain~time+I(time^2),random=~time,subject="ID",data=d.fab)

## Cubic model
m1c<-hlme(fixed=pain~time+I(time^2)+I(time^3),random=~time,subject="ID",data=d.fab)
```

Estimate the model with more than one class (G=2)

```
## Linear model
m2ag<-gridsearch(hlme(fixed=pain~time,random=~time,mixture=~time,subject="ID",ng=2,data=d.fab),rep = 100, maxiter = 30
, minit = m1a)
```

```r
## Quadratic model
m2bg<-gridsearch(hlme(fixed=pain~time+I(time^2),random=~time,mixture=~time+I(time^2), ng=2, subject="ID",data=d.fab),r
ep = 100, maxiter = 30, minit = m1b)

## Cubic model
m2cg <- gridsearch(hlme(fixed=pain~time+I(time^2)+I(time^3),random=~time,mixture=~time+I(time^2)+I(time^3), ng=2, subj
ect="ID",data=d.fab),rep =100,maxiter = 30, minit = m1c)
```

Estimate the model with more than one class (G=3)

```r
## Linear model
m3ag<-gridsearch(hlme(fixed=pain~time,random=~time,mixture=~time,subject="ID",ng=3,data=d.fab),rep = 100, maxiter = 30
, minit = m1a)

## Quadratic model
m3bg<-gridsearch(hlme(fixed=pain~time+I(time^2),random=~time,mixture=~time+I(time^2), ng=3, subject="ID",data=d.fab),r
ep = 100, maxiter = 30, minit = m1b)

## Cubic model
m3cg<-gridsearch(hlme(fixed=pain~time+I(time^2)+I(time^3),random=~time,mixture=~time+I(time^2)+I(time^3), ng=3, subjec
t="ID",data=d.fab, returndata = TRUE), rep=100, maxiter=30, minit=m1c)
```

Estimate the model with more than one class (G=4)

```r
## Linear model
m4ag<-gridsearch(hlme(fixed=pain~time,random=~time,mixture=~time,subject="ID",ng=4,data=d.fab), rep=100, maxiter=30, m
init=m1a)

## Quadratic model
m4bg<-gridsearch(hlme(fixed=pain~time+I(time^2),random=~time,mixture=~time+I(time^2), ng=4, subject="ID",data=d.fab),
rep=100, maxiter=30, minit = m1b)

## Cubic model
m4cg<-gridsearch(hlme(fixed=pain~time+I(time^2)+I(time^3),random=~time,mixture=~(time+I(time^2)+I(time^3)), ng=4, subj
ect="ID",data=d.fab, returndata = TRUE),rep=100, maxiter=30, minit=m1c)
```

Estimate the model with more than one class (G=5)

```
## Linear model
m5ag<-gridsearch(hlme(fixed=pain~time,random=~time,mixture=~time,subject="ID",ng=5,data=d.fab), rep=100, maxiter=30, m
init=m1a)

## Quadratic model
m5bg<-gridsearch(hlme(fixed=pain~time+I(time^2),random=~time,mixture=~time+I(time^2), ng=5, subject="ID",data=d.fab),
rep=100, maxiter=30, minit = m1b)

## Cubic model
m5cg<-gridsearch(hlme(fixed=pain~time+I(time^2)+I(time^3),random=~time,mixture=~(time+I(time^2)+I(time^3)), ng=5, subj
ect="ID",data=d.fab, returndata = TRUE),rep=100, maxiter=30, minit=m1c)
```

Model comparisons

```
## Model comparison
summarytable <-data.frame(summarytable(m1a,m1b,m1c,m2ag,m2bg,m2cg,m3ag,m3bg,m3cg,m4ag,m4bg,m4cg,m5ag,m5bg,m5cg, which=
c("G","loglik","conv","npm","AIC","BIC","SABIC","entropy","%class")))
summarytable
```

Additional model comparisons

```
## Model comparisons extended
LCTMcompare(m2cg,m3cg, m4cg, m5cg)

## Posterior probabilites
postprob(m2cg)
postprob(m3cg)
postprob(m4cg)
postprob(m5cg)

##LRM LRT calculations
calc_lrt(175,null_ll=-1379.305,null_param = 8, null_classes = 1, alt_ll = -1353.726, alt_param = 16, alt_classes = 2)
calc_lrt(175,null_ll=-1353.726,null_param = 16, null_classes = 2, alt_ll = -1343.763, alt_param = 21, alt_classes = 3)
calc_lrt(175,null_ll=-1343.763 ,null_param = 21, null_classes = 3, alt_ll = -1327.933, alt_param = 26, alt_classes = 4
```

```
)
calc_lrt(175,null_ll=-1327.933,null_param = 26, null_classes = 4, alt_ll = -1320.368, alt_param = 31, alt_classes = 5)
```

Supplementary Material 4 (S4)

Table S4: Sensitivity analysis of baseline characteristics

Variable	Drop-outs	Retained	p-value
Age	38.1	39.8	0.468
Female (%)	17.7	33.1	0.128
Lower educational level (%)	13.5	25.1	0.164
No work (%)	4	2.8	0.048
≤ moderate QoL (%)	14.9	25.7	0.103
Pain (NRS)	4.7	4.5	0.567
Daily frequent pain (%)	19.1	48.0	0.804
≥1 previous LBP episode (%)	22.0	55.5	0.689
Referred pain (%)	14.5	34.1	1
Treatment due to LBP (%)	19.0	44.6	0.918
LBP medication (%)	13.3	31.2	1
Imaging (%)	4.5	14.9	0.553
Disability (ODI)	20.3	15.6	0.014
Illness Perception: Timeline (IPQ)	2.25	2.36	0.520
Illness perception: Control (IPQ)	1.76	1.74	0.852
Endurance behavior (AEQ)	2.16	2.72	0.007
Avoidance behavior (AEQ)	2.9	3.61	0.017
Pain vigilance (PVAQ)	35.5	37.4	0.376
Depression (DASS21)	6.12	4.95	0.346
Stress (DASS21)	9.21	8.88	0.795
Anxiety (STAI-S)	40.0	36.8	0.101
Risk stratification (%, SBT), ≥ medium risk	9.6	20.4	0.513

Numbers are means (standard deviations) of participants unless stated otherwise.

QoL = Quality of life; NRS = Numeric Rating Scale; LBP = Low Back Pain; ODI = Oswestry Disability Index;
IPQ = Illness Perception Questionnaire; AEQ = Avoidance Endurance Questionnaire;
PVAQ = Pain Vigilance and Awareness Questionnaire; DASS21 = Depression Anxiety Stress Scale;
STAI-S = State Trait Anxiety Inventory – State, SBT = Start Back Tool
